# Validation of reference genes for use in untreated bovine fibroblasts

**DOI:** 10.1038/s41598-021-89657-8

**Published:** 2021-05-13

**Authors:** T. Toorani, P. M. Mackie, G. F. Mastromonaco

**Affiliations:** 1 Reproductive Sciences, Toronto Zoo, Scarborough, ON M1B 5K7 Canada; 2 Department of Biomedical Sciences, Ontario Veterinary College, University of Guelph, Guelph, ON N1G 2W1 Canada

**Keywords:** Cell biology, Molecular biology, Gene expression analysis

## Abstract

Proper normalization of RT-qPCR data is pivotal to the interpretation of results and accuracy of scientific conclusions. Though different approaches may be taken, normalization against multiple reference genes is now standard practice. Genes traditionally used and deemed constitutively expressed have demonstrated variability in expression under different experimental conditions, necessitating the proper validation of reference genes prior to utilization. Considering the wide use of fibroblasts in research and scientific applications, it is imperative that suitable reference genes for fibroblasts of different animal origins and conditions be elucidated. Previous studies on bovine fibroblasts have tested limited genes and/or samples. Herein, we present an extensive study investigating the expression stability of 16 candidate reference genes across 7 untreated bovine fibroblast cell lines subjected to controlled conditions. Data were analysed using various statistical tools and algorithms, including geNorm, NormFinder, BestKeeper, and RefFinder. A combined use of *GUSB* and *RPL13A* was determined to be the best approach for data normalization in untreated bovine fibroblasts.

## Introduction

The inherent properties of fibroblasts render them ideal cellular models for numerous research and scientific applications. Fibroblasts can be retrieved non-invasively and from a multitude of tissues, and are easily cultured and maintained in vitro^1^. Fibroblast culture and cryopreservation techniques are well-established, straightforward, and do not require specialized protocols even among different taxa^1,2^. Historically, fibroblast cell lines established from human and animal tissues have been used to improve our understanding of disease pathogenesis^3^, wound healing^4,5^, and normal fibroblast physiology. More recently, fibroblasts have been used as donor cells in somatic cell nuclear transfer (SCNT)^6,7^, and induced pluripotent stem cell applications (iPSC)^8–10^, which present powerful tools for disease modeling in vitro^11–16^, personalized and regenerative medicine, oncogenic applications^17^, and wildlife conservation^1^, among others. In many of these studies, describing transcriptional changes of key regulatory genes within the fibroblast has been crucial to the complete understanding of the cellular mechanisms underpinning their function. As a measure of transcriptional dynamics, quantification of mRNA abundance via reverse transcription quantitative real-time polymerase chain reaction (RT-qPCR) has become standard practice across many disciplines, owing to its theoretical and logistical simplicity^18^. However, while RT-qPCR permits the quantification of small amounts of nucleic acid and the detection of minute variability, including single copy differences^19^, it also presents a potential pitfall to this technique, unless proper normalization steps are taken. Accurate data analysis and interpretation following RT-qPCR are contingent upon suitable normalization methods to control for potential errors introduced throughout the multi-step process, including normalization to an internal reference gene (RG)^20^. Further, the Minimum Information for Publication of Quantitative Real-Time PCR Experiments (MIQE) guidelines recommend the use of multiple internal RGs for data normalization^18^, owing to the resolution of the data being defined by the stability of the reference genes under the given experimental conditions^20^.

The most commonly used “classical” RGs, including β-actin (*ACTB*), glyceraldehyde-3-phosphate dehydrogenase (*GAPDH*), hypoxanthine–guanine phosphoribosyl transferase (*HPRT*) and 18S ribosomal RNA (*18S rRNA*), are carryovers from references used in Northern blotting, RNase protection and conventional RT-PCR assays, which were suitable for these non- and semi-quantitative techniques where qualitative changes were evaluated^20^. However, the advent of quantitative techniques such as RT-qPCR should have instigated the evaluation of more suitable normalization approaches^20^. Instead, many investigators continue to use these classical RGs, assuming consistent expression without adequate experimental validation. For example, according to Chapman and Waldenström, *ACTB* and *GAPDH* continue to be the most widely used RGs, with 62% of studies using one of these genes as the single normalizing RG, of which only 8% did the due diligence of evaluating stability prior to use^21^. This is despite evidence showing that the expression stability of both *ACTB*^22–26^ and *GAPDH*^24,27–29^ are not impervious to experimental conditions. A further examination of RG stability validation studies showed a significant (P < 0.001) and inverse relationship between the number of RGs screened and the probability that *ACTB*, *GAPDH*, or *18S rRNA* were selected for normalization; where the likelihood of these three genes being selected is significantly decreased when more RGs are tested^21^. The importance of proper RG selection has been demonstrated by numerous studies reporting significant differences in results owing to normalization against RGs of varying stability^23,30,31^. It is unlikely that any universal RGs exist, and it is therefore important for the suitability of RGs to be experimentally validated for a given set of samples and conditions prior to use.

Optimal RGs have been reported for RT-qPCR use in various bovine cells^25,26,29,32–34^, including fibroblasts^31,35^. Interestingly, Zhou et al. reported larger variation when only one RG (*ACTB*) was used to calculate target gene expression compared to two RGs^31^. However, limitations in these studies, including small sample size (n = 1)^35^, number of RGs tested (5)^31^, and the use of at most two RG determination algorithms^31^, may have impacted the identification of optimal RGs for bovine fibroblast cells. The current study addresses a gap in knowledge concerning suitable RGs for use in RT-qPCR studies investigating untreated bovine fibroblast cell lines. We evaluated the expression stability of 16 candidate RGs across 7 untreated bovine fibroblast cell lines grown under controlled conditions and standardization by morphology and growth kinetics. Candidate genes were selected based on an extensive review of the literature (Supplementary Table S1) and included RGs previously described in fibroblast and/or bovine studies, as well as the classical RGs (Table 1). Special consideration was given to select genes from various pathways and functional classes to avoid co-regulation. Data were analysed using the most common RG determination methods: geNorm^36^, NormFinder^37^, BestKeeper^38^, and RefFinder^39^, which integrates the algorithms of the first three methods, as well as delta Ct^40^. The algorithms for each differ and, as such, their combined application should improve confidence in the selection of ideal candidate RGs when there is congruence. To our knowledge, this is the most extensive validation of RGs for use in untreated bovine fibroblasts undertaken to date.

**Table 1:** Overview and details of candidate RGs evaluated in this study.

Gene	Name	Function/pathway	NCBI accession no	Qiagen catalog no
*ACTB*	Actin, beta	Cytoskeletal structural protein	NM_173979.3	PPB00173A-200
*B2M*	Beta-2-microglobulin	Beta chain of MHC class 1 molecule	NM_173893.3	PPB00031A-200
*GAPDH*	Glyceraldehyde-3-phosphate dehydrogenase	Glycolysis and gluconeogenesis enzyme	NM_001034034.2	PPB00298A-200
*GUSB*	Glucuronidase, beta	Catalysis of complex carbohydrates breakdown	NM_001083436.1	PPB06553A-200
*HMBS*	Hydroxymethylbilane synthase	Heme biosynthetic pathway	NM_001046207.1	PPB06519A-200
*HPRT1*	Hypoxanthine phosphoribosyltransferase 1	Purine synthesis in salvage pathway	NM_001034035.2	PPB00330A-200
*HSP90AB1*	Heat shock 90 kDa protein 1, beta	Molecular chaperone	NM_001079637.1	PPB14507A-200
*PPIA*	Peptidylprolyl isomerase A	Protein folding	NM_178320.2	PPB00426A-200
*RAD50*	Radiation sensitive 50	DNA double-stranded break repair protein	NM_001206868.1	PPB15504A-200
*RPL13A*	Ribosomal protein L13a	Ribosome structural constituent; protein synthesis	NM_001076998.2	PPB14550A-200
*RPS18*	Ribosomal protein S18	Ribosomal protein, component of 40S subunit	NM_001033614.2	PPB01408A-200
*SDHA*	Succinate dehydrogenase complex, subunit A	Electron transporter in TCA cycle and respiratory chain	NM_174178.2	PPB00460A-200
*SF3A1*	Splicing factor 3a, subunit 1	pre-mRNA splicing; as a component of pre-catalytic spliceosome "B" complexes	NM_001081510.1	PPB02067A-200
*TBP*	TATA box binding protein	TATA box binding protein, general RNA polymerase II transcription factor	NM_001075742.1	PPB06797A-200
*UBC*	Ubiquitin C	Protein modifier; attaches to lysine	NM_001206307.2	PPB01883A-200
*YWHAZ*	Tyrosine 3-monooxygenase/tryptophan 5-monooxygenase activation protein zeta	Signal transduction via binding to phospherine-containing proteins	NM_174814.2	PPB01343A-200

## Results

### Range of fluctuation of RGs determined by box-and-whiskers plot

**Figure 1:**
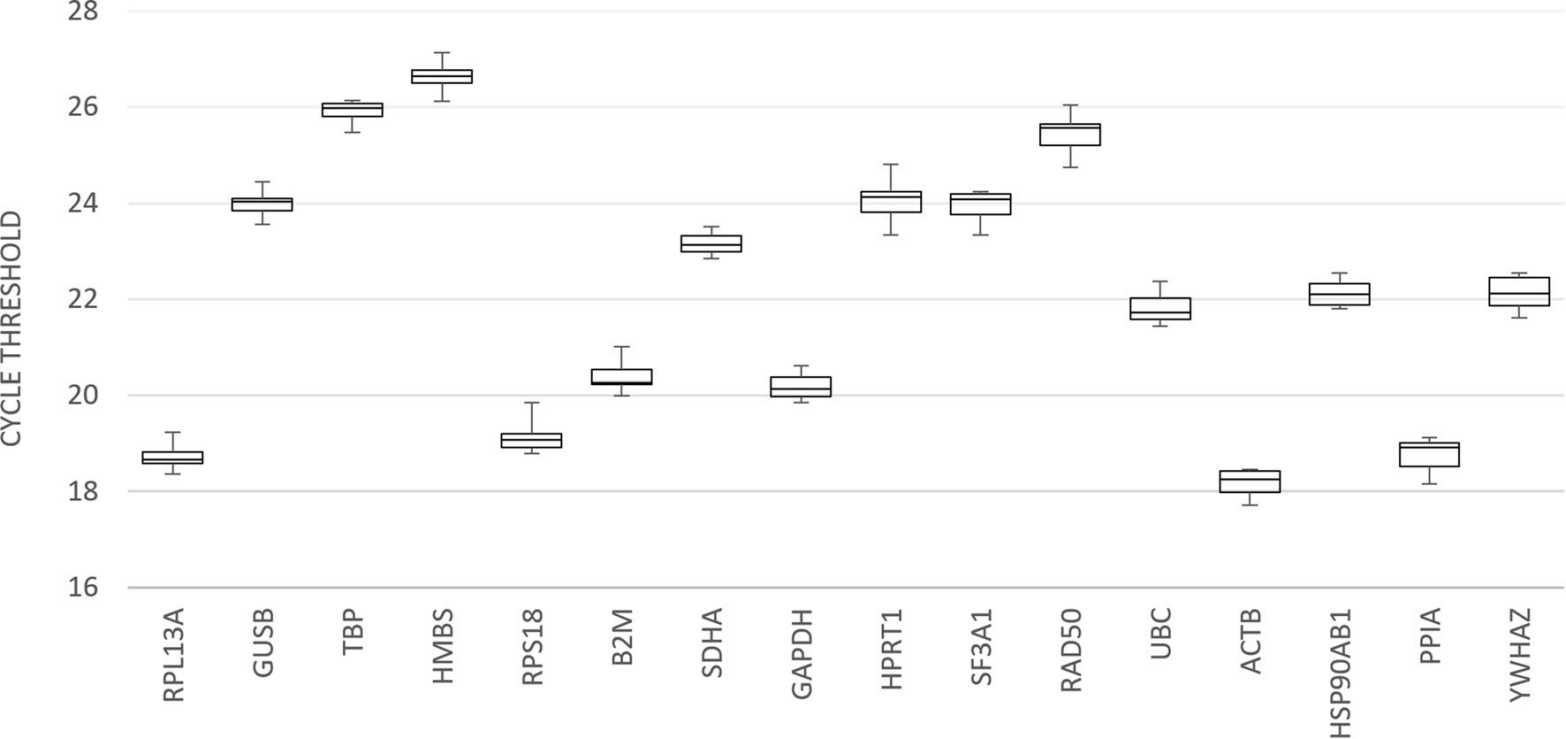
Range of fluctuation of candidate RG Ct values. Box-and-whiskers plot illustrating range of fluctuation of candidate RG Ct values across fibroblast cell lines (n = 7) in order (from left to right) of increasing interquartile range. Each cell line was reverse transcribed in triplicate and combined, and subsequently assayed in technical triplicates. Mean of technical replicates were used in this analysis. The first quartile, median, and third quartile are indicated by the lower limit of the box, the line within the box, and the upper limit of the box, respectively. The whiskers represent the minimum and maximum values. Data represent one independent experiment.

An extensive evaluation of the literature describing the use of RGs, including those specific to fibroblast and/or bovine cells, culminated in the selection of 16 candidate RGs for inclusion in this study. Following RT-qPCR, gene expression variability (Ct) was assessed for each of the candidate genes (Fig. 1). As a measure of stability, the range of fluctuation of each gene was determined by finding the difference between 25 and 75th percentiles, the interquartile range. *YWHAZ*, *PPIA* and *HSP90AB1* displayed the greatest variability, with ranges of fluctuation of 0.5798, 0.4909, and 0.4500 respectively, while the most stable genes were *RPL13A*, *GUSB* and *TBP* with ranges of 0.2368, 0.2442, and 0.2691, respectively.

### geNorm analysis of RG stability

**Figure 2:**
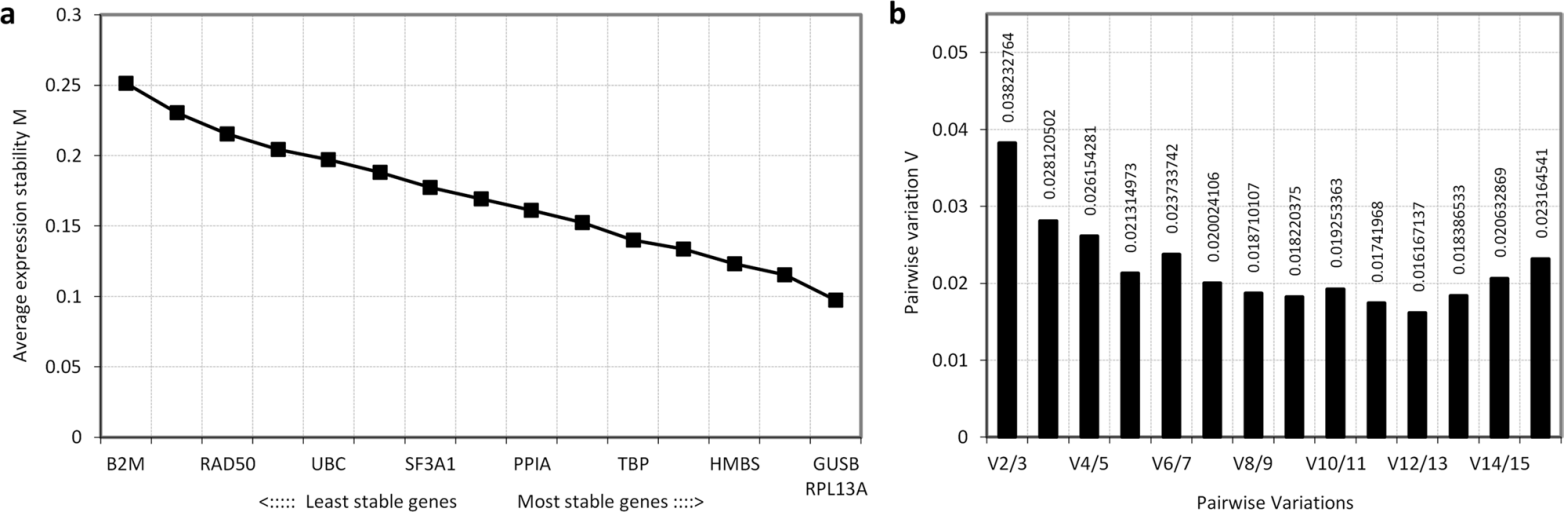
geNorm candidate RG output. Results of (**a**) the average stepwise exclusion analysis to determine genes with highest stability expressed as average expression stability (M) values, and (**b**) pairwise variation (V) values, to determine the optimal number of RGs. Consensus for M and V value cut-off is 0.5 and 0.15, respectively. Sixteen candidate RGs were assessed across n = 7 cell lines (reverse transcribed in triplicate and combined, and subsequently assayed in technical triplicates). Calculated relative quantity (RQ) of mean Ct values were used as input. Data represent one independent experiment.

The stability of the candidate RGs was analysed using four statistical methods. The algorithm, geNorm, is a comprehensive tool, performing an initial measure of expression stability (M) value assessment for all genes, followed by a stepwise exclusion analysis, wherein the least stable gene is eliminated, and the remaining genes are reassessed for M values. Although M values for all genes fell within range for inclusion (M < 0.5), *B2M*, *HPRT1*, and *RAD50* had the lowest stability while *GUSB*, *RPL13A*, and *SDHA* had the highest stability (Fig. 2a). Pairwise variation (V) values were also calculated to determine the suitable number of RGs required for untreated bovine fibroblast gene expression studies using RT-qPCR (Fig. 2b). Low pairwise variation (V) value for V2/3 = 0.0382 indicated that the two most stable RGs (*RPL13A* and *GUSB*) were suitable for data normalization and that adding a third reference gene would not provide additional value^41^. Though geNorm shows bias in selecting co-regulated genes, *RPL13A* and *GUSB* are not co-regulated (see Supplementary Tables S2 and S3), further corroborating their suitability as reference genes.

### NormFinder analysis of RG stability

Gene stability rankings were compared between the Microsoft Excel plug-in and R version of NormFinder (see Supplementary Table S4). While the former generated stability values, the latter computed GroupSD values for individual genes and combinations of two genes to only two decimal places, precluding the determination of gene rankings with certainty. However, rankings of both versions generally coincided: based on the output, *B2M*, *HPRT1*, and *RAD50* showed the lowest stability, while *HMBS*, *GUSB*, and *TBP* had the highest stability. The most stable combination of genes was *GAPDH* and *YWHAZ* (0.03), followed closely by *ACTB* and *SF3A1*, *GAPDH* and *SF3A1*, *GUSB* and *PPIA*, and *HMBS* and *RPL13A* (0.04) (Table 2).

**Table 2:** NormFinder candidate RG combination stability output.

Gene1	Gene2	GroupSD	RANK
*GAPDH*	*YWHAZ*	0.03	1
*ACTB*	*SF3A1*	0.04	2
*GAPDH*	*SF3A1*	0.04	2
*GUSB*	*PPIA*	0.04	2
*HMBS*	*RPL13A*	0.04	2
*ACTB*	*HMBS*	0.05	6
*GAPDH*	*SDHA*	0.05	6
*PPIA*	*TBP*	0.05	6
*ACTB*	*GUSB*	0.06	9
*ACTB*	*TBP*	0.06	9
*GAPDH*	*GUSB*	0.06	9
*GUSB*	*RPL13A*	0.06	9
*PPIA*	*SF3A1*	0.06	9
*RPL13A*	*SF3A1*	0.29	14
*PPIA*	*SDHA*	0.35	15

### BestKeeper analysis of RG stability

BestKeeper generated a set of descriptive statistics (Table 3), where genes falling short of certain cut-offs (standard deviation [± Ct] > 1 and/or standard deviation [± x-fold] > 2) were omitted from the subsequent BestKeeper index calculation that determines coefficients or correlation [r] and p values (Table 4). All genes satisfied the initial criterion and were kept in the subsequent analysis. Based on both standard deviation values (± Ct/ ± x-fold), *YWHAZ*, *PPIA* and *SF3A1* displayed the lowest stability, while *TBP*, *SDHA* and *RPL13A* had the highest stability. Using the coefficient of correlation [r] value, *GAPDH*, *SF3A1* and *RPL13A* displayed the lowest stability while *ACTB*, *YWHAZ* and *PPIA* showed the highest stability.

**Table 3:** BestKeeper candidate RG descriptive statistics, including standard deviation [± Ct], which is used as a cut-off for subsequent BestKeeper Index calculation.

	*ACTB*	*GAPDH*	*GUSB*	*HMBS*	*PPIA*	*RPL13A*	*SDHA*	*SF3A1*	*TBP*	*YWHAZ*
Geometric mean [Ct]	18.19	20.18	23.97	26.64	18.75	18.71	23.17	23.94	25.92	22.13
Arithmetic mean [Ct]	18.20	20.18	23.97	26.64	18.75	18.71	23.17	23.94	25.92	22.13
Min [Ct]	17.73	19.85	23.55	26.13	18.13	18.35	22.88	23.34	25.48	21.61
Max [Ct]	18.49	20.60	24.46	27.11	19.08	19.23	23.48	24.24	26.14	22.56
Standard deviation [± Ct]	0.25	0.23	0.21	0.22	0.29	0.20	0.19	0.28	0.18	0.31
Coefficient of variance [% Ct]	1.36	1.14	0.86	0.82	1.54	1.06	0.83	1.16	0.71	1.40
Min [x-fold]	− 1.38	− 1.26	− 1.33	− 1.43	− 1.53	− 1.28	− 1.23	− 1.52	− 1.35	− 1.44
Max [x-fold]	1.23	1.33	1.41	1.39	1.26	1.43	1.24	1.24	1.17	1.35
Standard deviation [± x-fold]	1.19	1.17	1.15	1.16	1.22	1.15	1.14	1.21	1.14	1.24

**Table 4:** BestKeeper Index calculation, used in determining coefficients of correlation [r] and p values for candidate RGs.

BestKeeper vs	*ACTB*	*GAPDH*	*GUSB*	*HMBS*	*PPIA*	*RPL13A*	*SDHA*	*SF3A1*	*TBP*	*YWHAZ*
Coeff. of corr. [r]	0.974	0.869	0.937	0.940	0.954	0.883	0.906	0.872	0.947	0.955
*p*-value	0.001	0.011	0.002	0.002	0.001	0.008	0.005	0.011	0.001	0.001

### RefFinder analysis of RG stability

**Figure 3:**
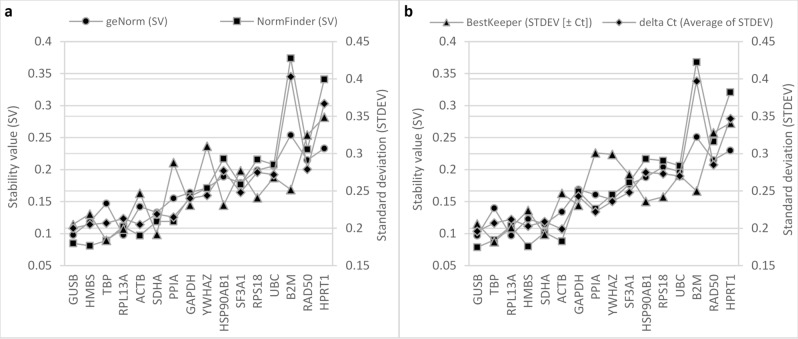
RefFinder candidate RG output. RefFinder results using (**a**) median, and (**b**) mean Ct values of technical replicates. Genes showing the highest stability, as determined by RefFinder’s comprehensive ranking, are ordered left to right on the x-axis. Individual results from embedded algorithms (geNorm, NormFinder, BestKeeper, and delta Ct) are also illustrated. Lower stability values (SV) and standard deviation (STDEV) are indicative of more stably expressed genes. Sixteen candidate RGs were assessed across n = 7 cell lines, which were reverse transcribed in triplicate and combined, and consequently assayed in technical triplicates. Data represent one independent experiment.

RefFinder assesses gene stability based on the algorithms of geNorm, NormFinder, BestKeeper, and delta Ct, and generates a comprehensive ranking based on the geometric mean of ranking values derived from the four other algorithms. As RefFinder does not specify input requirements, results derived from the use of median and mean Ct values were compared. Remarkably, for all four imbedded algorithms and the comprehensive ranking, the top four most stable genes were the same between the median Ct and mean Ct datasets, albeit in slightly different orders for geNorm, NormFinder and the comprehensive rankings. Each algorithm generated slightly different outcomes (Fig. 3). For the median Ct dataset, results from geNorm, NormFinder, and delta Ct identified genes *B2M*, *HPRT1*, and *RAD50* as the least stable, while BestKeeper determined *HPRT1*, *RAD50*, and *YWHAZ* as least stable. RGs with the highest stability were as follows: geNorm determined the combination of *GUSB* and *RPL13A*, and *HMBS*; NormFinder identified *HMBS*, *GUSB*, and *TBP*; delta Ct identified *GUSB*, *ACTB*, and *HMBS*; and BestKeeper determined *TBP*, *SDHA*, and *RPL13A* as the most suitable. Overall, *HPRT1*, *RAD50*, and *B2M* had the poorest rankings, while *GUSB*, *HMBS*, and *TBP* showed the highest stability. With respect to the mean Ct dataset, the least stable genes were determined by geNorm, NormFinder, and delta Ct to be *B2M*, *HPRT1*, and *RAD50*, while BestKeeper flagged *HPRT1*, *RAD50*, and *PPIA*. The highest stability was shown by the combination of *GUSB*/*RPL13A*, and *SDHA* according to geNorm; *GUSB*, *HMBS*, and *ACTB* based on NormFinder; *TBP*, *SDHA*, and *RPL13A* according to BestKeeper; and finally, *GUSB*, *ACTB*, and *HMBS* based on delta Ct. The comprehensive ranking determined *HPRT1*, *RAD50*, and *B2M* as the least stable genes and *GUSB*, *TBP*, and *RPL13A* as the most stable genes. For detailed results from both datasets, see Supplementary Table S5.

### Overview of rankings from algorithms

The results generated by the box-and-whiskers analysis, geNorm, NormFinder, RefFinder, and BestKeeper have been summarized in Table 5.

**Table 5:** Compilation of rankings of candidate RGs by all statistical tools and algorithms.

Candidate RGs	Box-and-whiskers	geNorm	NormFinder		RefFinder (median Ct/mean Ct)					*BestKeeper (Excel plug-in)	
		Excel plug-in	Excel plug-in	R-based	geNorm	NormFinder	BestKeeper	delta Ct	Comprehensive ranking	Standard deviation of Ct	Coefficient of correlation [r]
*GUSB*	2	1	2	1	1/1	2/1	4/4	1/1	1/1	4	6
*HMBS*	4	4	1	1	3/4	1/2	5/5	3/3	2/4	5	5
*TBP*	3	6	3	3	6/6	3/4	1/1	4/4	3/2	1	4
*RPL13A*	1	1	5	5	1/1	5/6	3/3	5/6	4/3	3	8
*ACTB*	13	5	4	4	5/5	4/3	9/9	2/2	5/6	7	1
*SDHA*	7	3	7	6	4/3	7/5	2/2	7/5	6/5	2	7
*PPIA*	15	8	6	6	7/8	6/7	13/14	6/7	7/8	9	3
*GAPDH*	8	9	8	8	8/9	8/9	7/6	8/9	8/7	6	10
*YWHAZ*	16	7	9	9	9/7	9/8	14/13	9/8	9/9	10	2
*HSP90AB1*	14	11	13	12	11/11	13/13	6/7	13/13	10/11	–	–
*RPS18*	5	13	12	12	12/13	12/12	8/8	12/12	12/12	–	–
*UBC*	12	12	11	11	13/12	11/11	11/11	11/11	13/13	–	–
*SF3A1*	10	10	10	10	10/10	10/10	12/12	10/10	11/10	8	9
*RAD50*	11	14	14	14	14/14	14/14	15/15	14/14	15/15	–	–
*B2M*	6	16	16	16	16/16	16/16	10/10	16/16	14/14	–	–
*HPRT1*	9	15	15	15	15/15	15/15	16/16	15/15	16/16	–	–

*The six genes most frequently ranked with the lowest stability were omitted from analysis using BestKeeper, which is limited to evaluating only 10 genes.

## Discussion

The selection of suitable RGs for RT-qPCR data normalization is pivotal to the integrity and reliability of results^18^. The present study aimed to identify stably expressed RGs for use in RT-qPCR normalization of untreated bovine fibroblasts by assessing a panel of 16 candidate RGs using numerous algorithms and statistical tools.

Several considerations were made in selecting the most optimal RG in this instance. Overall, rankings by different algorithms were generally in agreement, except for BestKeeper, which consistently generated different outcomes. The differences in BestKeeper outcomes, as compared to other RG determination algorithms, may be explained, at least partly, by the use of raw Ct values as input (as compared to the RQ values required by geNorm and NormFinder). Rydbirk and colleagues offer an alternate explanation, referring to the BestKeeper Index, a tool unique to the eponymous software, as the source of this discrepancy in outcome^42^. The BestKeeper Index is calculated from the geometric mean of all RGs satisfying the initial exclusion criterion (standard deviation [± Ct] > 1) and is subsequently used to generate Pearson correlation (r) values (coefficient of correlation) for each RG in comparison to this index, thereby determining expression stability. Setting aside BestKeeper results, some genes displayed consistently poor rankings (*B2M*, *HPRT1*, *HSP90AB1*, *RAD50*, RSP18, *SF3A1*, and *UBC*), while others consistently showed higher expression stability and the tightest range of Ct values (*GUSB*, *HMBS*, *TBP*, and *RPL13A*). A summary of rankings can be found in Table 5. Regarding similarities between the same algorithms used in different software, both versions of NormFinder were largely in agreement. RefFinder outputs and those of their original counterparts were also highly similar (other than BestKeeper, since RefFinder ranks RGs according to SG, and not the BestKeeper Index). Results from the mean and median were comparable, with small differences observed mostly between genes that do not show much difference in stability ranking.

Considering each algorithm uses a different approach and evaluates different parameters when determining RG expression stability, it is expected that variations in the results will be observed, though many studies have reported general consensus between algorithms^43^. As such, difficulties arise when trying to reconcile different rankings and several considerations must be made in selecting optimal RG(s), including the (i) overlap of rankings by various algorithms, (ii) strengths and weaknesses of each algorithm, as applicable to the experimental design of each study, and (iii) use of different complementary tools for merging results into a comprehensive ranking. GeNorm’s pairwise correlation is known to be a robust algorithm for studies with small sample sizes but shows bias towards selecting RGs that are co-regulated. NormFinder’s model-based approach confers the advantage of differentiating intragroup variation from intergroup variation, and as such, is a suitable tool for the assessment of RGs in experiments with different sample groups, but requires larger sample sizes (> 8) in comparison to geNorm^37,43,44^. Different tools have been reported by investigators to merge results of different algorithms into a comprehensive ranking, the most well-known of which is RefFinder^39^. Concerns have been raised about the validity of this software mainly based on the fact that RefFinder does not integrate primer efficiencies in calculations, and that its BestKeeper output is solely based on the initial standard deviation [± Ct] values and not the BestKeeper Index. Another limitation of RefFinder is that results of the four methods are not weighed given the unavailability of their cut-offs and applicable weights^45^. Lastly, cycle threshold coefficient of variation (Ct CV%)^46^ can also be used to determine comprehensive ranking whereby a lower Ct CV% is indicative of higher stability and is one of the parameters found in the initial descriptive statistics generated by BestKeeper. Though this presents a simple approach in assessing RG stability, Ct CV% should be considered carefully, as it is not always inversely correlated to the degree of stability of a given gene^47^. Selection of the most suitable algorithm for a given experimental design must take into account the different strengths and weaknesses as described above. In our case, given our small sample size, geNorm was the most suitable algorithm, but the results from all algorithms were taken into consideration before selecting the optimal RGs.

The panel of genes evaluated showed good stability (e.g. all genes satisfied geNorm’s M value criterion of 0.5, NormFinder’s standard deviation [± Ct] of 1, and geNorm’s V value criterion of 0.15), compared to the range of stabilities seen for candidate RGs assessed in other studies^26,32^. This is to be expected due to the set of standardized untreated fibroblasts used, with average M values between 0.2–0.5 typically being observed when assessing candidate RGs across a homogeneous sample set^41^. *GUSB* showed the most consistently stable expression amongst all RGs, ranking first or second in 12 of the 16 rankings. The use of a single RG, however, is discouraged and can create variability in the expression of the target genes independent of experimental treatments^31^, including more than threefold deviation from true values in 25% of cases, and at least sixfold in 10% of cases^36^. The appropriate number of RGs required must be experimentally validated. GeNorm determines the number and combinations of genes showing stability while NormFinder evaluates all possible pairs of genes. In our study, according to geNorm, the best approach was a combined use of *GUSB* and *RPL13A*, which was corroborated by both versions of NormFinder, where this combination, though tied for 9th in rankings, showed greater stability than the number 1 ranked individual genes *HMBS* and *GUSB* (Group SD of 0.06 vs 0.08, respectively). Further investigation of *GUSB* and *RPL13A* revealed no-coregulation between these two genes, an important consideration when selecting RGs^37,43,44^. Individually, *GUSB* and *RPL13A* show high and medium/medium–high expression stabilities, respectively. It is their combined expression (geometric mean of expression) that shows high stability and was therefore determined as the optimal approach for data normalization in untreated bovine fibroblasts. These two genes also display the smallest range of Ct values (Fig. 1). Previous RG validation in bovine fibroblasts concluded the combined use of *ACTB* and *YWHAZ* as the most suitable data normalization approach (using geNorm and NormFinder), and showed that data normalization against one RG (*ACTB*; which showed high expression stability within the genes assessed) can yield results deviating from true values^31^. However, this study only assessed five candidate genes using RNA from treated (serum-starved cells) and co-regulation between *ACTB* and *YWHAZ* did not appear to be considered^31^.

The information provided here addresses a gap in knowledge concerning suitable reference genes for use in RT-qPCR studies of untreated bovine fibroblast cell lines, a commonly studied species. This type of validation, though essential, is costly and time consuming. To this end, the current study was designed as the most extensive RG validation in untreated fibroblast cell lines to date. Our results provide targeted information for future RT-qPCR studies, alleviating the researcher of the burden of repeating the validation. For example, the results presented herein were used to normalize gene expression when comparing inherent expression between various bovine fibroblast cells lines^48^. The limitation of this paper, however, is that any deviations applied in future studies (i.e. different experimental treatments, cell culture conditions), will warrant novel validation of suitable reference genes. However, investigators can use the reference genes found suitable herein as a starting point, or broaden their evaluation to include any of the 16 candidate genes carefully selected from the literature and further tested for co-regulation. Finally, we also highlight the varying strengths and weaknesses of the most published algorithms, and recommend using a multitude of approaches when evaluating gene stability to ensure accurate RT-qPCR normalization under your given experimental conditions.

We conclude that the combined use of *GUSB* and *RPL13A* presents the best approach in normalizing RT-qPCR data in untreated bovine fibroblasts. Though these genes have been less frequently used for normalization, they have previously been shown as suitable RGs in human adipose tissue- and Wharton’s Jelly-derived mesenchymal stromal cells (*RPL13A*)^24^, human nasal epithelium cells (*GUSB*)^49^, human papillary thyroid carcinoma (*GUSB*)^50^, human dermal fibroblasts and mammary epithelial cells (*GUSB*)^51^, rat bone mesenchymal stem cell-derived neuronal cells (*RPL13A*)^52^, and treated human ovarian cancer cells (*RPL13A*)^53^.

## Materials and methods

### Tissue processing and fibroblast cell culture

Ears from healthy, age-matched (< 30 mos.) Angus bulls were removed post-mortem at the local abattoir, covered with sterile saline and transported to the lab for processing within 4 h of collection. Tissue samples (n = 7) were processed, and fibroblast cells cultured as previously described^54^ with minor modifications. Previous experiments have shown comparable outcome between sample sizes of n = 11 and n = 6^43^. Given the homogeneity of the samples (i.e. untreated bovine fibroblasts), it was deemed that n = 7 was adequate for the determination of suitable reference genes. All materials and reagents were obtained from Thermo Fisher Scientific (Mississauga, ON, Canada) unless otherwise noted. Briefly, tissue samples spanning the entire thickness of the ears were collected using an 8 mm biopsy punch, the skin was discarded, and the remaining cartilage was manually dissected into 1 mm^2^ pieces before being collagenase-digested (Sigma-Aldrich, Oakville, ON, Canada) for 5 h at 38.5 °C and 5% CO_2_. Cells and tissue fragments were pelleted by centrifugation at 200 rcf for 5 min, resuspended in Dulbecco's Modified Eagle’s Medium (DMEM; Sigma-Aldrich, Oakville, ON, Canada) supplemented with 20% fetal bovine serum (FBS; Sigma-Aldrich, Oakville, ON, Canada) and 1% antibiotic–antimycotic (ABAM), and cultured at 38.5 °C and 5% CO_2_ in 25 cm^2^ tissue culture flasks. Media were partially and fully replaced on days 4 and 6, respectively, until confluency, at which point cells were passaged at a dilution of 1:4 into DMEM containing 10% FBS and 1% penicillin–streptomycin (P/S). After 2 passages (P3), confluent cell lines were trypsin-harvested (0.25% trypsin–EDTA), diluted 1:4 in freezing medium containing DMEM supplemented with 20% FBS, 10% dimethyl sulfoxide (DMSO; Sigma-Aldrich, Oakville, ON, Canada), and 1% P/S, and cryopreserved. For consistency, only one ear was processed per day and all steps were conducted by the same operator. Cell lines that did not reach confluency within 9-days from the initiation of culture or contained > 10% cells with abnormal morphology (assessed visually) were discarded. All cell lines were authenticated for cell type homogeneity, chromosome normality (2n = 60) and sex, as described by Toorani et al.^48^. Inclusion and exclusion criteria were pre-established.

### RNA extraction and cDNA synthesis

Fibroblasts were thawed, cultured to confluency (P4), pelleted, and frozen at − 80 °C for RNA extraction using a Total RNA Purification Kit (Norgen Biotek Corp., Thorold, ON, Canada) following manufacturer’s instructions, including an on-column gDNA removal step (RNase-Free DNase I kit, Norgen Biotek Corp., Thorold, ON, Canada). Isolated RNA was quantified using the Qubit RNA HS Assay Kit. RNA integrity was assessed visually (28S and 18S rRNA) following separation on 1% E-GEL, EX-Agarose gels using the Invitrogen E-Gel Power Snap Electrophoresis System.

Reverse transcription (RT) was performed using 1000 ng of RNA, and in accordance with, Norgen Biotek Corp.’s TruScript First Strand cDNA Synthesis Kit (Thorold, ON, Canada). Samples were stored at − 20 °C before proceeding to reverse transcription quantitative real-time PCR (RT-qPCR).

### Selection of candidate genes

A panel of 16 candidate RGs was selected based on an extensive review of the literature (see Supplementary Table S1). Classical RGs were chosen, as well as others used or validated for use in bovine or fibroblast cells. Special consideration was taken to select genes from different functional classes and pathways to avoid co-regulation, which may skew the output of some of the algorithms used in RG analysis^37,43^. Custom RT^2^ Profiler PCR arrays were ordered from Qiagen (Cat No./ID 330,171, Toronto, ON, Canada) and used as per manufacturer’s instructions. Selected RGs were further assessed for co-regulation using the Database of Gene Co-Regulation (dGCR; www.dGCR.org)^55^ based on data available for humans, mice, and rats from Affymetrix, Illumina, and Agilent (see Supplementary Tables S2 and S3).

### Reverse Transcription Quantitative Real-time PCR (RT-qPCR)

Custom 96-well (0.2 ml) RT^2^ Profiler PCR arrays pre-coated with the primers for the 16 candidate RGs were purchased from Qiagen and RT-qPCR was performed following manufacturer’s instructions on a QuantStudio3 Real-Time PCR System (Thermo Fisher Scientific, Applied Biosystems, Foster City, CA, USA). Briefly, a master-mix containing 4.5 ng of cDNA and 12.5 µl of RT^2^ SYBR Green ROX Mastermix (Qiagen, Toronto, ON, Canada) per reaction was prepared for each sample and loaded into the array plates in triplicate for each of the 16 genes (48 reactions per sample). Plates were briefly centrifuged. Thermocycling profile consisted of an initial cycle of 95 °C for 10 min, followed by 40 cycles at 95 °C for 15 s and 1 min at 60 °C. Dissociation curves (95 °C for 15 s, 60 °C for 1 min, and 60 °C to 95 °C at 0.15 °C s^-1^ with signal acquisition) were evaluated to confirm primer specificity. To correct for inter-plate variation one sample was repeated on all plates. All Qiagen RT^2^ primers have been previously validated and optimized to work at uniform and near perfect efficiency to allow for simultaneous analysis of multiple genes in arrays. Primers are designed using proprietary algorithms and are subjected to rigorous testing for high performance: the average amplification efficiency of a representative set of assays for 4000 genes used in RT^2^ Arrays was shown to be 99%, with a 95% confidence interval about the mean between 90 and 110%^56^. Therefore, a base of 2 was used for relative quantity calculations^38,43,57^.

### Data analysis

Ct values and baseline corrections to account for inter-plate gene expression variation were calculated using Thermo Fisher Connect (Thermo Fisher Scientific, Applied Biosystems, Foster City, CA, USA). Relative quantification values (RQ) were calculated using RQ = E^(min Ct − sample Ct)^ where *E*, the amplification efficiency, is 2 (assume 100% efficiency for commercially designed primers)^38,43,57^, *min Ct* is the sample with the lowest Ct value (highest expression) and *sample Ct* is the Ct of each sample. This will result in a set of relative quantities between 1 and 0, where the sample with the lowest Ct is 1. The calculation (*min Ct-sample Ct)* is also referred to as ΔCt.

The calculated stability of candidate RGs and their relative ranking was compared across four statistical methods, geNorm, NormFinder, RefFinder, and BestKeeper. Calculated RQ of mean Ct values were input in geNorm (Version 3.5) to determine expression stability (M) for each gene, as well as the optimal number of RGs required by comparing pairwise variation (V)^36^. Accepted cut-off values are M < 0.5 and V < 0.15; when the Vn/Vn + 1 ratio is less than 0.15, it indicates that the optimum number is n^36^. Two versions of NormFinder, Microsoft Excel add-in (Version 0.953) and NormFinder for R (Version 5, 2015–01-05), were evaluated using RQ values of median Ct as input for both versions to calculate stability for individual genes and gene combinations (https://moma.dk/normfinder-software)^37^. RefFinder (https://www.heartcure.com.au/reffinder)^39^ evaluates expression stability based on the algorithms of geNorm, NormFinder, BestKeeper, and delta Ct, generating a comprehensive ranking based on the geometric mean of ranking values. Input data format (median or mean Ct values) was not specified and so results from both methods were evaluated. Finally, BestKeeper (Version 1) requires input of raw median Ct values to determine the expression stability by using pairwise correlation analysis of all pairs of candidate genes^38^. Standard deviations, coefficients of correlation [r] and p-values determined for each gene are used as measures of RG stability, with coefficients of correlation being the superior metric. BestKeeper allows for the assessment of up to 10 candidate RGs, therefore, the 6 genes most often ranking poorly according to the box-and-whiskers assessment and algorithms geNorm, NormFinder and RefFinder (*UBC*, *RAD50*, *HPRT1*, *B2M*, *HSP90AB1*, and *RPS18*) were omitted from this analysis.

## Supplementary Information

Supplementary Table S1.

Supplementary Table S2.

Supplementary Table S3.

Supplementary Table S4.

Supplementary Table S5.

## Data Availability

Datasets generated and analysed during the current study are available in the figshare repository, http://dx.doi.org/10.6084/m9.figshare.1266254064.

## References

[1] Mastromonaco GF, González-Grajales LA, Filice M, Comizzoli P, Holt WV, Brown JL, Comizzoli P (2014). Somatic cells, stem cells, and induced pluripotent stem cells: How do they now contribute to conservation?. Reproductive Sciences in Animal Conservation Advances in Experimental Medicine and Biology.

[2] Freshney RI (2010). Culture of Animal Cells: A Manual of Basic Technique and Specialized Applications.

[3] Villegas J, McPhaul M (2005). Establishment and culture of human skin fibroblasts. Curr. Protoc. Mol. Biol..

[4] des Jardins-Park HE, Foster DS, Longaker MT (2018). Fibroblasts and wound healing: An update. Regen. Med..

[5] Larson BJ, Longaker MT, Lorenz HP (2010). Scarless fetal wound healing: A basic science review. Plast. Reconstr. Surg..

[6] Gouveia C, Huyser C, Egli D, Pepper MS (2020). Lessons learned from somatic cell nuclear transfer. Int. J. Mol. Sci..

[7] Campbell KH (2007). Somatic cell nuclear transfer: Past, present and future perspectives. Theriogenology.

[8] Takahashi K (2007). Induction of pluripotent stem cells from adult human fibroblasts by defined factors. Cell.

[9] Yu J (2007). Induced pluripotent stem cell lines derived from human somatic cells. Science.

[10] Takahashi K, Yamanaka S (2006). Induction of pluripotent stem cells from mouse embryonic and adult fibroblast cultures by defined factors. Cell.

[11] Gu BW (2015). Impaired telomere maintenance and decreased canonical WNT signaling but normal ribosome biogenesis in induced pluripotent stem cells from X-linked dyskeratosis congenita patients. PLoS ONE.

[12] Freitas BCG (2012). Stem cells and modeling of autism spectrum disorders. Exp. Neurol..

[13] Chang T (2011). Brief report: Phenotypic rescue of induced pluripotent stem cell-derived motoneurons of a spinal muscular atrophy patient. Stem Cells.

[14] Marchetto MC (2010). A model for neural development and treatment of Rett syndrome using human induced pluripotent stem cells. Cell.

[15] Dimos JT (2008). Induced pluripotent stem cells generated from patients with ALS can be differentiated into motor neurons. Science.

[16] Park IH (2008). Disease-specific induced pluripotent stem cells. Cell.

[17] Navarro AM, Susanto E, Falk A, Wilhelm M (2018). Modeling cancer using patient-derived induced pluripotent stem cells to understand development of childhood malignancies. Cell Death Discov..

[18] Bustin SA (2009). The MIQE guidelines: Minimum information for publication of quantitative real-time PCR experiments. Clin. Chem..

[19] Palmer S (2003). New real-time reverse transcriptase-initiated PCR assay with single-copy sensitivity for human immunodeficiency virus type 1 RNA in plasma. J. Clin. Microbiol..

[20] Huggett J, Dheda K, Bustin S, Zumla A (2005). Real-time RT-PCR normalisation; strategies and considerations. Genes Immun..

[21] Chapman JR, Waldenström J (2015). With reference to reference genes: A systematic review of endogenous controls in gene expression studies. PLoS ONE.

[22] Banfi F, Colombini A, Perucca Orfei C, Parazzi V, Ragni E (2018). Validation of reference and identity-defining genes in human mesenchymal stem cells cultured under unrelated fetal bovine serum batches for basic science and clinical application. Stem Cell Rev. Rep..

[23] Panina Y, Germond A, Masui S, Watanabe TM (2018). Validation of common housekeeping genes as reference for qPCR gene expression analysis during iPS reprogramming process. Sci. Rep..

[24] Amable PR, Teixeira MVT, Carias RBV, Granjeiro JM, Borojevic R (2013). Identification of appropriate reference genes for human mesenchymal cells during expansion and differentiation. PLoS ONE.

[25] Spalenza V (2011). Identification of internal control genes for quantitative expression analysis by real-time PCR in bovine peripheral lymphocytes. Vet. J..

[26] Goossens K (2005). Selection of reference genes for quantitative real-time PCR in bovine preimplantation embryos. BMC Dev. Biol..

[27] Roy JG, McElhaney JE, Verschoor CP (2020). Reliable reference genes for the quantification of mRNA in human T-cells and PBMCs stimulated with live influenza virus. BMC Immunol..

[28] Sugden K, Pariante CM, McGuffin P, Aitchison KJ, D’Souza UM (2010). Housekeeping gene expression is affected by antidepressant treatment in a mouse fibroblast cell line. J. Psychopharmacol..

[29] Pérez R, Tupac-Yupanqui I, Dunner S (2008). Evaluation of suitable reference genes for gene expression studies in bovine muscular tissue. BMC Mol. Biol..

[30] Nielsen S (2018). Optimal reference genes for normalization of qPCR gene expression data from proton and photon irradiated dermal fibroblasts. Sci. Rep..

[31] Zhou W (2009). Transcript levels of several epigenome regulatory genes in bovine somatic donor cells are not correlated with their cloning efficiency. Cloning Stem Cells.

[32] Anstaett OL, Brownlie J, Collins ME, Thomas CJ (2010). Validation of endogenous reference genes for RT-qPCR normalisation in bovine lymphoid cells (BL-3) infected with Bovine Viral Diarrhoea Virus (BVDV). Vet. Immunol. Immunop..

[33] Lisowski P, Pierzchała M, Gościk J, Pareek CS, Zwierzchowski L (2008). Evaluation of reference genes for studies of gene expression in the bovine liver, kidney, pituitary, and thyroid. J. Appl. Genet..

[34] Emam M, Thompson-Crispi K, Mallard B (2005). The effect of immunological status, in-vitro treatment and culture time on expression of eleven candidate reference genes in bovine blood mononuclear cells. BMC Immunol..

[35] Zhou W (2008). Global gene expression analysis of bovine blastocysts produced by multiple methods. Mol. Reprod. Dev..

[36] Vandesompele J (2002). Accurate normalization of real-time quantitative RT-PCR data by geometric averaging of multiple internal control genes. Genome Biol..

[37] Andersen CL, Jensen JL, Ørntoft TF (2004). Normalization of real-time quantitative reverse transcription-PCR data: A model-based variance estimation approach to identify genes suited for normalization, applied to bladder and colon cancer data sets. Cancer Res..

[38] Pfaffl MW, Tichopad A, Prgomet C, Neuvians TP (2004). Determination of stable housekeeping genes, differentially regulated target genes and sample integrity: BestKeeper—Excel-based tool using pair-wise correlations. Biotechnol. Lett..

[39] Xie F, Xiao P, Chen D, Xu L, Zhang B (2012). miRDeepFinder: A miRNA analysis tool for deep sequencing of plant small RNAs. Plant Mol. Biol..

[40] Silver N, Best S, Jiang J, Thein SL (2006). Selection of housekeeping genes for gene expression studies in human reticulocytes using real-time PCR. BMC Mol. Biol..

[41] Hellemans J, Vandesompele J, Biassoni R, Raso A (2014). Selection of reliable reference genes for RT-qPCR analysis. Quantitative Real-Time PCR. Methods in Molecular Biology (Methods and Protocols).

[42] Rydbirk R (2016). Assessment of brain reference genes for RT-qPCR studies in neurodegenerative diseases. Sci. Rep..

[43] De Spiegelaere W (2015). Reference gene validation for RT-qPCR, a note on different available software packages. PLoS ONE.

[44] Mehta R (2010). Validation of endogenous reference genes for qRT-PCR analysis of human visceral adipose samples. BMC Mol. Biol..

[45] Wu H, Taki FA, Zhang Y, Dobbins DL, Pan X (2014). Evaluation and identification of reliable reference genes for toxicological study in Caenorhabditis elegans. Mol. Biol. Rep..

[46] Caradec J (2010). ‘Desperate house genes’: The dramatic example of hypoxia. Br. J. Cancer.

[47] Wang X (2018). Identification and validation of appropriate reference genes for qRT-PCR analysis in *Corynebacterium glutamicum*. FEMS Microbiol. Lett..

[48] Toorani T, Mackie PM, Mastromonaco GF (2021). Investigating markers of reprogramming potential in somatic cell lines derived from matched donors. Cell Reprogr..

[49] Masvidal L (2012). *GUSB* and *ATP2B4* are suitable reference genes for *CFTR* gene expression data normalization in nasal epithelium cells. J. Cyst. Fibros..

[50] Razavi SA (2019). Validation of reference genes for normalization of relative qRT-PCR studies in papillary thyroid carcinoma. Sci. Rep..

[51] González-Bermúdez L, Anglada T, Genescà A, Martín M, Terradas M (2019). Identification of reference genes for RT-qPCR data normalisation in aging studies. Sci. Rep..

[52] He Y-X, Zhang Y, Yang Q, Wang C, Su G (2015). Selection of suitable reference genes for reverse transcription-quantitative polymerase chain reaction analysis of neuronal cells differentiated from bone mesenchymal stem cells. Mol. Med. Rep..

[53] Bian Z (2014). RPL13A as a reference gene for normalizing mRNA transcription of ovarian cancer cells with paclitaxel and 10-hydroxycamptothecin treatments. Mol. Med. Rep..

[54] Mastromonaco GF, Perrault SD, Betts DH, King WA (2006). Role of chromosome stability and telomere length in the production of viable cell lines for somatic cell nuclear transfer. BMC Dev. Biol..

[55] Williams G (2015). Database of gene co-regulation (dGCR): A web tool for analysing patterns of gene co-regulation across publicly available expression data. J. Genom..

[56] Quellhorst, G. & Rulli, S. *A Systematic Guideline for Developing the Best Real-Time PCR Primers: Lessons Learned from Designing Assays for More Than 14,000 Genes*. https://www.qiagen.com/us/resources/download.aspx?id=d6191d0e-701b-4eb1-bafa-d7ab7677875f&lang=en (2012).

[57] Kałużna M, Kuras A, Puławska J (2017). Validation of reference genes for the normalization of the RT-qPCR gene expression of virulence genes of *Erwinia amylovora* in apple shoots. Sci. Rep..

[58] Bionaz M, Loor JJ (2007). Identification of reference genes for quantitative real-time PCR in the bovine mammary gland during the lactation cycle. Physiol. Genomics.

[59] Rekawiecki R, Rutkowska J, Kotwica J (2012). Identification of optimal housekeeping genes for examination of gene expression in bovine corpus luteum. Reprod. Biol..

[60] Rekawiecki R, Kowalik MK, Kotwica J (2013). Validation of housekeeping genes for studying differential gene expression in the bovine myometrium. Acta Vet. Hung..

[61] Robert C (2002). Quantification of housekeeping transcript levels during the development of bovine preimplantation embryos. Biol. Reprod..

[62] Robinson TL, Sutherland IA, Sutherland J (2007). Validation of candidate bovine reference genes for use with real-time PCR. Vet. Immunol. Immunopathol..

[63] Ross PJ, Wang K, Kocabas A, Cibelli JB (2010). Housekeeping gene transcript abundance in bovine fertilized and cloned embryos. Cell. Reprogram..

[64] Toorani T, Mackie PM, Mastromonaco GF (2021). Figshare.

