# Supplementary material for: Validation of reference genes for use in untreated bovine fibroblasts
Source: Sci Rep. 2021 May 13;11:10253. doi: 10.1038/s41598-021-89657-8 (PMC8119449; doi:10.1038/s41598-021-89657-8)
Supplement: Supplementary file 1 — Supplementary Table S1. [file 41598_2021_89657_MOESM1_ESM.docx]

Validation of reference gene for use in untreated bovine fibroblasts

Toorani T., Mackie P. M. & Mastromonaco G. F.

**Supplementary Table S1 - Literature survey of reference gene selection experiments.** Summary of the literature surveyed to select candidate reference genes (RGs) for validation in untreated bovine fibroblasts. In the last column (Optimal RGs [by algorithm]) a forward slash denotes that the geometric mean of the RGs should be used for suitable data normalization, while “+” denotes that any of the listed RGs were found to be suitable for data normalization in their respective experiments.

| **Author** | **Species** | **Cell/tissue** | **Candidate genes** | **Optimal RGs**  **[by algorithm]** |
| --- | --- | --- | --- | --- |
| Amable *et al.*^24^ | Human | Bone marrow-derived mesenchymal stromal cells (BM-MSC); Adipose tissue (AT-MSC); Umbilical cord Wharton's Jelly-derived mesenchymal stromal cells (WJ-MSC); Dermal fibroblasts (DF) | *ACTB* | *HPRT1* (BM-MSC)  *RPL13A* (AT-MSC)  *RPL13A* (WJ-MSC)  *HPRT1* (DF)  [geNorm]  *GAPDH* (WJ-MSC)  *HPRT1* (DF)  [NormFinder]  *RPL13A* + *B2M* (BM-MSC)  *GAPDH* + *RPL13A* (AT-MSC)  *RPL13A* + *B2M* (WJ-MSC) *B2M* + *RPL13A* (DF)  [BestKeeper] |
|  |  |  | *B2M* |  |
|  |  |  | *GAPDH* |  |
|  |  |  | *HPRT1* |  |
|  |  |  | *RPL13A* |  |
| Anstaett *et al.*^32^ | Bovine | Lymphoid cells | *ACTB* | *B2M*/*YWHAZ* + *GAPDH*  [geNorm]  *GAPDH* + *ACTB* + *HPRT*  [NormFinder]  *B2M* + *YWHAZ* + *SDHA*  [BestKeeper] |
|  |  |  | *B2M* |  |
|  |  |  | *GAPDH* |  |
|  |  |  | *HPRT* |  |
|  |  |  | *PPIA* |  |
|  |  |  | *RPL4* |  |
|  |  |  | *SDHA* |  |
|  |  |  | *YWHAZ* |  |
| Banfi *et al.*^22^ | Human | Bone marrow-derived mesenchymal stem cells (BMMSC); Umbilical cord-blood-derived mesenchymal stem cells (CBMSC); Mesenchymal stem cells (MSC) | *18S rRNA* | *YWHAZ/UBC*, V2/3 = 0.036 (BMMSC)  *RPLP0/EF1A*, V2/3 = 0.027 (CBMSC)  *EF1A/TBP*, V2/3 = 0.034 (MSC)  [geNorm]  *YWHAZ* + *UBC* + *EF1A* (BMMSC)  *RPLP0* + *EF1A* + *PPIA, RPL13A, YWHAZ* (last three tied) (CBMSC)  *EF1A* + *TBP* + *UBC*, *RPL13A* (last two tied) (MSC)  [NormFinder] |
|  |  |  | *ACTB* |  |
|  |  |  | *B2M* |  |
|  |  |  | *EF1A* |  |
|  |  |  | *GAPDH* |  |
|  |  |  | *GUSB* |  |
|  |  |  | *PPIA* |  |
|  |  |  | *RPL13A* |  |
|  |  |  | *RPLP0* |  |
|  |  |  | *TBP* |  |
|  |  |  | *UBC* |  |
|  |  |  | *YWHAZ* |  |
| Bionaz & Loor^58^ | Bovine | Mammary gland tissue | *ACTB* | *UXT/RPS9* (V2/3 = 0.081) + *RPS15* (V3/4 = 0.067) [geNorm] |
|  |  |  | *GAPDH* |  |
|  |  |  | *GTP* |  |
|  |  |  | *ITGB4BP* |  |
|  |  |  | *MRPL39* |  |
|  |  |  | *RPS9* |  |
|  |  |  | *RPS15* |  |
|  |  |  | *RPS23* |  |
|  |  |  | *UXT* |  |
| Emam *et al.*^34^ | Bovine | BMC | *18S rRNA* | *CTBP1* + *RAD50*  [Statistical analysis with SAS qPCR] |
|  |  |  | *24S rRNA* |  |
|  |  |  | *ACTB* |  |
|  |  |  | *B2M* |  |
|  |  |  | *CTBP1* |  |
|  |  |  | *GAPDH* |  |
|  |  |  | *MDM4* |  |
|  |  |  | *PPIA* |  |
|  |  |  | *RAD50* |  |
|  |  |  | *SDHA* |  |
|  |  |  | *YWHAZ* |  |
| Goossens *et al.*^26^ | Bovine | Embryos | *18S rRNA* | *GAPDH*/*SDHA*/*YWHAZ* (V3/4 ~ 0.23*)  [geNorm] *lowest V value (V4/5 ~0.33) |
|  |  |  | *ACTB* |  |
|  |  |  | *GAPDH* |  |
|  |  |  | *H2A* |  |
|  |  |  | *HPRT1* |  |
|  |  |  | *SDHA* |  |
|  |  |  | *TBP* |  |
|  |  |  | *YWHAZ* |  |
| Lisowski *et al.*^33^ | Bovine | Liver, kidney, pituitary, and thyroid | *ACTB* | *GAPDH* + *YWHAZ* (kidney)  *ACTB* + *TBP* + *YWHAZ* + *GAPDH* + *HPRTI* + *SDHA* (liver)  *GAPDH* + *SDHA* (pituitary)  *TBP* + *HPRTI* (thyroid)  [geNorm] |
|  |  |  | *GAPDH* |  |
|  |  |  | *HPRT1* |  |
|  |  |  | *SDHA* |  |
|  |  |  | *TBP* |  |
|  |  |  | *YWHAZ* |  |
| Nielsen *et al.*^30^ | Human | Fibroblasts | *ACTB* | *PUM1* + *CALM2* + *MRPL19*  [RefFinder, comprehensive]  *IPO8* + *MRPL19* + *PSMC4*  [RefFinder, geNorm]  *PUM1/CALM2*  [RefFinder, NormFinder]  *RPL37A* + *PMM1*  [RefFinder, BestKeeper]  *PUM1*  [RefFinder, deltaCt] |
|  |  |  | *ACTR3* |  |
|  |  |  | *B2M* |  |
|  |  |  | *CALM2* |  |
|  |  |  | *CHCHD1* |  |
|  |  |  | *GAPDH* |  |
|  |  |  | *GUSB* |  |
|  |  |  | *HMBS* |  |
|  |  |  | *HPRT1* |  |
|  |  |  | *IPO8* |  |
|  |  |  | *MRPL19* |  |
|  |  |  | *NDFIP1* |  |
|  |  |  | *PMM1* |  |
|  |  |  | *POLR2A* |  |
|  |  |  | *PSMC4* |  |
|  |  |  | *PPIA* |  |
|  |  |  | *PUM1* |  |
|  |  |  | *RPL37A* |  |
|  |  |  | *RPLP0* |  |
|  |  |  | *SF3A1* |  |
|  |  |  | *TBP* |  |
|  |  |  | *TFRC* |  |
| Panina *et al.*^23^ | Murine | Fibroblasts (embryonic) | *ACTB* | *ATP5F1/PGK1* + *GAPDH*  [geNorm]  *ATP5F1* + *PGK1* + *GUSB*  [NormFinder]  *ATP5F1* + *PGK1* + *GAPDH*  [BestKeeper]  *ATP5F1* + *GAPDH* + *PGK1*  [deltaCt]  *ATP5F1* + *PGK1* + *GAPDH*  [RefFinder, comprehensive] |
|  |  |  | *ATP5F1* |  |
|  |  |  | *B2M* |  |
|  |  |  | *GAPDH* |  |
|  |  |  | *GUSB* |  |
|  |  |  | *HPRT* |  |
|  |  |  | *PGK1* |  |
|  |  |  | *PPIA* |  |
|  |  |  | *RPS18* |  |
|  |  |  | *TBP* |  |
|  |  |  | *TFRC* |  |
|  |  |  | *YWHAZ* |  |
| Pérez *et al.*^29^ | Bovine | Longissimus dorsi muscle | *18S rRNA* | *EEF1A2/SF3A1* (V2/3 = 0.130) + *HMBS* (V3/4 = 0.101)  [geNorm]  *EEF1A2* + *HMBS* + *CASC3*  [NormFinder]  *HMBS* + *EEF1A2* + *18s rRNA*  [BestKeeper] |
|  |  |  | *ACTB* |  |
|  |  |  | *B2M* |  |
|  |  |  | *CASC3* |  |
|  |  |  | *EEF1A2* |  |
|  |  |  | *GAPDH* |  |
|  |  |  | *HMBS* |  |
|  |  |  | *RP2* |  |
|  |  |  | *SF3A1* |  |
|  |  |  | *UBC* |  |
| Rekawiecki *et al.*^59^ | Bovine | Corpus luteum | *18S rRNA* | *C2ORF29/TBP* (V2/3 < 0.11) + *SUZ12* (V3/4 < 0.10)  [geNorm]  *C2ORF29* + *SUZ12* + *TBP*  [NormFinder] |
|  |  |  | *ACTB* |  |
|  |  |  | *B2M* |  |
|  |  |  | *C2ORF29* |  |
|  |  |  | *GAPDH* |  |
|  |  |  | *HPRT1* |  |
|  |  |  | *MRPL12* |  |
|  |  |  | *SDHA* |  |
|  |  |  | *SF3A1* |  |
|  |  |  | *SUZ12* |  |
|  |  |  | *TBP* |  |
|  |  |  | *TUBB2B* |  |
|  |  |  | *ZNF131* |  |
| Rekawiecki *et al.*^60^ | Bovine | Myometrium | *18S rRNA* | *C2ORF29/TBP* (V2/3 = 0.15) + *TUBB2B* (V3/4 = 0.112)  [geNorm]  *C2ORF29* + *MRPL12* + *TBP*  [NormFinder] |
|  |  |  | *ACTB* |  |
|  |  |  | *B2M* |  |
|  |  |  | *C2ORF29* |  |
|  |  |  | *GAPDH* |  |
|  |  |  | *HPRT1* |  |
|  |  |  | *MRPL12* |  |
|  |  |  | *SDHA* |  |
|  |  |  | *SF3A1* |  |
|  |  |  | *SUZ12* |  |
|  |  |  | *TBP* |  |
|  |  |  | *TUBB2B* |  |
|  |  |  | *ZNF131* |  |
| Robert *et al.*^61^ | Bovine | Preimplantation embryo | *18S rRNA* | *H2A* |
|  |  |  | *ACTB* |  |
|  |  |  | *GAPDH* |  |
|  |  |  | *H2A* |  |
|  |  |  | *LMNB* |  |
|  |  |  | *Tubulin* |  |
|  |  |  | *U2snRNA* |  |
|  |  |  | *Ubiquitin* |  |
| Robinson *et al.*^62^ | Bovine | BMC | *18S rRNA* | *ACTB/GAPDH* + *ACTB*  [geNorm]  *GAPDH* + *ACTB/GAPDH*  [Normfinder]  *ACTB/GAPDH* + *ACTB*  [BestKeeper] |
|  |  |  | *ACTB* |  |
|  |  |  | *GAPDH* |  |
|  |  |  | *RPLP0* |  |
| Ross *et al.*^63^ | Bovine | Embryos | *ACTB* | H2A.2 and PPIA: consistent expression levels between IVF and SCNT embryos within any given developmental stage. Their expression varied across different developmental stages. |
|  |  |  | *GAPDH* |  |
|  |  |  | *GUSB* |  |
|  |  |  | *H2A.2* |  |
|  |  |  | *PPIA* |  |
|  |  |  | *RPL15* |  |
| Spalenza *et al.*^25^ | Bovine | Lymphocytes | *24S rRNA* | *PPIA/S24* (V2/3 = 0.087) + *YWAHZ* (V3/4 = 0.089)  [geNorm]  *YWAHZ* + *GAPDH* + *S24*  [NormFinder]  *YWHAZ* + *GAPDH* + *PPIA*  [BestKeeper] |
|  |  |  | *ACTB* |  |
|  |  |  | *GAPDH* |  |
|  |  |  | *HPRT1* |  |
|  |  |  | *PPIA* |  |
|  |  |  | *SDHA* |  |
|  |  |  | *YWHAZ* |  |
| Sugden *et al.*^28^ | Mouse | Fibroblasts | *18S rRNA* | *ATP5B/CYC1* (V2/3 = 0.09) + *B2M* (V3/4 = 0.10)  [geNorm]  *ATP5B* + *B2M* + *CYC1*  [NormFinder] |
|  |  |  | *ACTB* |  |
|  |  |  | *ATP5B* |  |
|  |  |  | *B2M* |  |
|  |  |  | *CANX* |  |
|  |  |  | *CYC1* |  |
|  |  |  | *EIF4A2* |  |
|  |  |  | *GAPDH* |  |
|  |  |  | *RPL13A* |  |
|  |  |  | *SDHA* |  |
|  |  |  | *UBC* |  |
|  |  |  | *YWHAZ* |  |
| Vandesompele *et al.*^36^ | Human | Neuroblastoma; Fibroblasts; Leukocytes; Bone marrow; | *ACTB* | *HPRT1/GAPDH/ SDHA/UBC* (V4/5 = 0.138) (Neuroblastoma)  *HPRT1/GAPDH/YWHAZ* (V3/4 = 0.109) (Fibroblast)  *UBC/YWHAZ/B2M* (V3/4 < 0.13) (Leukocyte)  *UBC/RPL13A/YWHAZ* (V3/4 < 0.10) (Bone marrow)  *SDHA/GAPDH/HMBS/HPRT1/TBP* (V5/6 = 0.148) (Normal pool)  [geNorm] |
|  |  |  | *B2M* |  |
|  |  |  | *GAPDH* |  |
|  |  |  | *HMBS* |  |
|  |  |  | *HPRT1* |  |
|  |  |  | *RPL13A* |  |
|  |  |  | *SDHA* |  |
|  |  |  | *TBP* |  |
|  |  |  | *UBC* |  |
|  |  |  | *YWHAZ* |  |
| Zhou *et al.*^31^ | Bovine | Fibroblasts | *ACTB* | *ACTB/YWHAZ*  [Genex software: geNorm and NormFinder] |
|  |  |  | *GAPDH* |  |
|  |  |  | *H2AFZ* |  |
|  |  |  | *RPS8* |  |
|  |  |  | *YWHAZ* |  |
| Zhou *et al.*^35^ | Bovine | Fibroblasts | Microarray | *RPS8/H2AFZ/YWHAZ*  [geNorm] |
