# Supplementary material for: Validation of reference genes for use in untreated bovine fibroblasts
Source: Sci Rep. 2021 May 13;11:10253. doi: 10.1038/s41598-021-89657-8 (PMC8119449; doi:10.1038/s41598-021-89657-8)
Supplement: Supplementary file 2 — Supplementary Table S2. [file 41598_2021_89657_MOESM2_ESM.docx]

Validation of reference gene for use in untreated bovine fibroblasts.

Toorani T., Mackie P. M. & Mastromonaco G. F.

**Supplementary Table S2 - Candidate Reference gene (RG) co-regulation assessment (based on data for human, mouse, and rat).** Overview of candidate reference gene co-regulation evaluated using the Database of Gene Co-Regulation (dGCR; [www.dGCR.org](http://www.dGCR.org)), as part of evaluation of suitable RGs.

| **Reference gene** | **Co-regulated RG in top 150** | **Rank (1-150)** | **dGCR score** | **Platforms** | **Correlation details** |
| --- | --- | --- | --- | --- | --- |
| ACTB | UBC | 8 | -750.24 | 16 | 0.92(-116.06) 0.98(-28.28) 0.81(-11.71) 0.67(-177.89) 0.18(-9.08) 0.46(-13.24) 0.45(-21.01) 0.94(-52.99) 0.73(-27.33) 0.82(-18.15) 0.82(-69.15) 0.83(-25.43) 0.94(-75.98) 0.66(-12.05) 1.00(-58.49) 0.86(-33.40) |
|  | YWHAZ | 11 | -628.04 | 16 | 0.86(-112.02) 0.98(-34.82) 0.81(-19.98) 0.48(-49.90) 0.64(-35.05) 0.70(-77.11) 0.88(-22.74) 0.72(-42.55) 0.51(-14.11) 0.65(-47.20) 0.84(-64.15) 0.47(-17.94) 0.82(-8.36) 0.87(-13.76) 0.97(-28.33) 0.95(-40.02) |
|  | PPIA | 29 | -485.79 | 14 | 0.81(-67.19) 0.93(-41.82) 0.98(-30.21) 0.57(-33.33) 0.49(-12.91) 0.12(-8.73) 0.80(-42.18) 0.78(-28.71) 0.74(-28.06) 0.88(-30.26) 0.42(-8.60) 0.89(-20.25) 1.00(-75.81) 0.90(-57.73) |
|  | B2M | 44 | -403.05 | 11 | 0.96(-145.56) 0.98(-34.98) 0.87(-13.92) 0.34(-14.59) 0.44(-21.71) 0.54(-52.26) 0.33(-13.96) 0.38(-9.29) 0.39(-17.74) 1.00(-66.39) 0.80(-12.65) |
|  | SDHA | 102 | -265.22 | 7 | 0.75(-17.99) 0.62(-15.15) 0.86(-102.79) 0.77(-9.56) 0.47(-16.01) 0.81(-76.61) 0.66(-27.11) |
| B2M | None of the candidate RGs listed |  |  |  |  |
| GAPDH | HSP90AB1 | 12 | -603.08 | 14 | 0.92(-176.00) 0.98(-51.31) 0.90(-52.57) 0.88(-15.91) 0.40(-25.67) 0.56(-10.92) 0.84(-47.16) 0.70(-25.14) 0.68(-17.13) 0.69(-16.43) 0.49(-12.08) 0.83(-47.16) 0.95(-18.42) 0.99(-87.18) |
|  | RPL13A | 18 | -524.53 | 12 | 0.87(-146.95) 0.99(-93.23) 0.99(-70.26) 0.95(-17.11) 0.46(-15.81) 0.65(-12.44) 0.48(-13.09) 0.91(-11.55) 0.91(-31.76) 1.00(-16.38) 0.96(-85.57) 0.14(-10.38) |
|  | PPIA | 30 | -494.52 | 12 | 0.74(-70.19) 0.93(-60.34) 0.91(-56.78) 0.93(-19.39) 0.12(-9.65) 0.94(-57.66) 0.40(-10.55) 0.71(-8.73) 1.00(-25.52) 0.96(-26.31) 0.99(-107.10) 0.33(-42.30) |
|  | UBC | 32 | -492.43 | 16 | 0.80(-117.40) 0.96(-43.97) 0.90(-31.28) 0.80(-8.97) 0.55(-27.03) 0.51(-12.66) 0.48(-16.11) 0.54(-12.03) 0.54(-25.63) 0.67(-21.81) 0.77(-21.16) 0.49(-10.58) 0.94(-18.92) 1.00(-9.42) 1.00(-105.46) 0.25(-10.00) |
|  | RPS18 | 50 | -416.5 | 10 | 0.98(-131.12) 0.99(-79.89) 0.99(-24.70) 0.94(-8.44) 0.71(-10.59) 0.53(-19.29) 0.76(-8.26) 0.86(-22.87) 1.00(-85.38) 0.31(-25.96) |
| GUSB | None of the candidate RGs listed |  |  |  |  |
| HMBS | None of the candidate RGs listed |  |  |  |  |
| HPRT1 | YWHAZ | 120 | -307.91 | 7 | 0.55(-66.90) 0.55(-24.76) 0.92(-25.15) 0.69(-27.04) 0.80(-63.47) 0.92(-25.15) 0.94(-75.44) |
| HSP90AB1 | YWHAZ | 6 | -1625.23 | 11 | 0.89(-527.04) 0.97(-420.21) 0.92(-291.63) 0.45(-17.65) 0.36(-10.56) 0.29(-8.39) 0.68(-14.94) 0.78(-17.27) 0.73(-65.78) 1.00(-8.01) 0.97(-243.75) |
|  | GAPDH | 68 | -603.08 | 14 | 0.92(-176.00) 0.98(-51.31) 0.90(-52.57) 0.88(-15.91) 0.40(-25.67) 0.56(-10.92) 0.84(-47.16) 0.70(-25.14) 0.68(-17.13) 0.69(-16.43) 0.49(-12.08) 0.83(-47.16) 0.95(-18.42) 0.99(-87.18) |
|  | RPL13A | 106 | -501.57 | 13 | 0.81(-140.98) 0.98(-55.87) 0.86(-78.29) 0.76(-40.70) 0.37(-12.28) 0.53(-25.24) 0.69(-15.74) 0.86(-25.26) 0.44(-10.44) 0.83(-13.37) 0.80(-9.81) 0.96(-60.18) 0.87(-13.41) |
| PPIA | GAPDH | 23 | -494.52 | 12 | 0.74(-70.19) 0.93(-60.34) 0.91(-56.78) 0.93(-19.39) 0.12(-9.65) 0.94(-57.66) 0.40(-10.55) 0.71(-8.73) 1.00(-25.52) 0.96(-26.31) 0.99(-107.10) 0.33(-42.30) |
|  | ACTB | 26 | -485.79 | 14 | 0.81(-67.19) 0.93(-41.82) 0.98(-30.21) 0.57(-33.33) 0.49(-12.91) 0.12(-8.73) 0.80(-42.18) 0.78(-28.71) 0.74(-28.06) 0.88(-30.26) 0.42(-8.60) 0.89(-20.25) 1.00(-75.81) 0.90(-57.73) |
|  | B2M | 37 | -460.51 | 9 | 0.55(-30.27) 0.87(-214.99) 0.64(-42.11) 0.77(-29.73) 0.61(-17.65) 0.89(-20.22) 0.39(-10.00) 0.96(-87.32) 0.32(-8.22) |
|  | YWHAZ | 77 | -380.28 | 10 | 0.54(-67.65) 0.81(-70.88) 0.62(-22.66) 0.51(-15.15) 0.41(-10.48) 0.79(-33.80) 0.60(-14.39) 0.91(-19.28) 0.93(-98.00) 0.64(-27.99) |
|  | RPS18 | 79 | -376.91 | 11 | 0.71(-50.81) 0.92(-45.21) 0.94(-14.87) 0.84(-19.60) 0.85(-16.87) 1.00(-14.53) 0.94(-13.68) 1.00(-18.99) 0.58(-10.31) 1.00(-95.10) 0.66(-76.94) |
|  | UBC | 81 | -366.98 | 11 | 0.23(-13.32) 0.80(-82.63) 0.53(-41.95) 0.38(-32.52) 0.87(-27.37) 0.45(-19.00) 0.73(-20.87) 0.35(-9.41) 0.88(-16.29) 0.99(-72.14) 0.68(-31.48) |
|  | RPL13A | 87 | -358.36 | 13 | 0.64(-79.29) 0.91(-37.06) 0.86(-33.99) 0.81(-23.09) 0.81(-10.24) 0.62(-9.04) 0.57(-11.21) 0.80(-15.07) 0.80(-12.39) 0.83(-9.61) 1.00(-24.32) 0.97(-71.42) 0.41(-21.63) |
|  | HSP90AB1 | 120 | -333.81 | 8 | 0.65(-60.37) 0.80(-98.75) 0.60(-40.52) 0.73(-11.92) 0.53(-8.56) 0.81(-15.46) 0.94(-69.05) 0.93(-29.18) |
| RAD50 | None of the candidate RGs listed |  |  |  |  |
| RPL13A | RPS18 | 25 | -1255.01 | 17 | 0.99(-303.37) 1.00(-110.88) 0.99(-74.51) 0.99(-119.72) 0.93(-77.82) 0.96(-24.19) 0.95(-130.31) 1.00(-23.28) 0.49(-34.85) 0.89(-63.76) 0.84(-26.45) 1.00(-46.21) 0.92(-12.65) 0.94(-19.29) 0.97(-43.77) 1.00(-109.93) 0.48(-34.02) |
|  | GAPDH | 120 | -524.53 | 12 | 0.87(-146.95) 0.99(-93.23) 0.99(-70.26) 0.95(-17.11) 0.46(-15.81) 0.65(-12.44) 0.48(-13.09) 0.91(-11.55) 0.91(-31.76) 1.00(-16.38) 0.96(-85.57) 0.14(-10.38) |
|  | HSP90AB1 | 129 | -501.57 | 13 | 0.81(-140.98) 0.98(-55.87) 0.86(-78.29) 0.76(-40.70) 0.37(-12.28) 0.53(-25.24) 0.69(-15.74) 0.86(-25.26) 0.44(-10.44) 0.83(-13.37) 0.80(-9.81) 0.96(-60.18) 0.87(-13.41) |
| RPS18 | RPL13A | 20 | -1255.01 | 17 | 0.99(-303.37) 1.00(-110.88) 0.99(-74.51) 0.99(-119.72) 0.93(-77.82) 0.96(-24.19) 0.95(-130.31) 1.00(-23.28) 0.49(-34.85) 0.89(-63.76) 0.84(-26.45) 1.00(-46.21) 0.92(-12.65) 0.94(-19.29) 0.97(-43.77) 1.00(-109.93) 0.48(-34.02) |
|  | B2M | 111 | -520.15 | 9 | 0.96(-153.62) 0.98(-47.29) 0.90(-18.29) 0.63(-52.90) 0.75(-103.70) 0.57(-13.52) 0.69(-31.67) 1.00(-82.41) 0.52(-16.75) |
|  | GAPDH | 141 | -416.5 | 10 | 0.98(-131.12) 0.99(-79.89) 0.99(-24.70) 0.94(-8.44) 0.71(-10.59) 0.53(-19.29) 0.76(-8.26) 0.86(-22.87) 1.00(-85.38) 0.31(-25.96) |
| SDHA | ACTB | 35 | -265.22 | 7 | 0.75(-17.99) 0.62(-15.15) 0.86(-102.79) 0.77(-9.56) 0.47(-16.01) 0.81(-76.61) 0.66(-27.11) |
| SF3A1 | None of the candidate RGs listed |  |  |  |  |
| TBP | None of the candidate RGs listed |  |  |  |  |
| UBC | ACTB | 1 | -750.24 | 16 | 0.92(-116.06) 0.98(-28.28) 0.81(-11.71) 0.67(-177.89) 0.18(-9.08) 0.46(-13.24) 0.45(-21.01) 0.94(-52.99) 0.73(-27.33) 0.82(-18.15) 0.82(-69.15) 0.83(-25.43) 0.94(-75.98) 0.66(-12.05) 1.00(-58.49) 0.86(-33.40) |
|  | GAPDH | 8 | -492.43 | 16 | 0.80(-117.40) 0.96(-43.97) 0.90(-31.28) 0.80(-8.97) 0.55(-27.03) 0.51(-12.66) 0.48(-16.11) 0.54(-12.03) 0.54(-25.63) 0.67(-21.81) 0.77(-21.16) 0.49(-10.58) 0.94(-18.92) 1.00(-9.42) 1.00(-105.46) 0.25(-10.00) |
|  | B2M | 15 | -448.84 | 10 | 0.78(-119.62) 0.90(-120.29) 0.40(-21.87) 0.61(-21.26) 0.65(-8.15) 0.40(-8.45) 0.44(-11.30) 0.75(-20.36) 1.00(-15.46) 1.00(-102.08) |
|  | RPL13A | 19 | -433.13 | 12 | 0.82(-138.04) 0.95(-49.66) 0.85(-29.75) 0.59(-18.41) 0.93(-32.46) 0.96(-30.89) 0.72(-18.16) 0.72(-14.19) 0.93(-14.40) 0.87(-13.95) 0.96(-60.09) 0.46(-13.13) |
|  | PPIA | 27 | -366.98 | 11 | 0.23(-13.32) 0.80(-82.63) 0.53(-41.95) 0.38(-32.52) 0.87(-27.37) 0.45(-19.00) 0.73(-20.87) 0.35(-9.41) 0.88(-16.29) 0.99(-72.14) 0.68(-31.48) |
|  | HSP90AB1 | 28 | -363.31 | 11 | 0.66(-99.87) 0.83(-71.79) 0.56(-31.72) 0.30(-8.84) 0.30(-17.26) 0.54(-11.04) 0.78(-12.40) 0.35(-8.41) 0.78(-18.24) 0.99(-72.22) 0.92(-11.52) |
|  | RPS18 | 40 | -328.04 | 9 | 0.90(-118.21) 0.98(-62.53) 0.50(-10.22) 0.90(-24.93) 0.79(-11.04) 0.75(-15.94) 0.79(-10.17) 0.99(-60.98) 0.50(-14.02) |
|  | YWHAZ | 44 | -313.55 | 10 | 0.62(-103.40) 0.88(-43.73) 0.44(-10.01) 0.41(-8.12) 0.79(-20.35) 0.49(-8.26) 0.74(-16.73) 0.52(-8.90) 0.96(-78.16) 0.64(-15.89) |
| YWHAZ | HSP90AB1 | 3 | -1625.23 | 11 | 0.89(-527.04) 0.97(-420.21) 0.92(-291.63) 0.45(-17.65) 0.36(-10.56) 0.29(-8.39) 0.68(-14.94) 0.78(-17.27) 0.73(-65.78) 1.00(-8.01) 0.97(-243.75) |
|  | ACTB | 83 | -628.04 | 16 | 0.86(-112.02) 0.98(-34.82) 0.81(-19.98) 0.48(-49.90) 0.64(-35.05) 0.70(-77.11) 0.88(-22.74) 0.72(-42.55) 0.51(-14.11) 0.65(-47.20) 0.84(-64.15) 0.47(-17.94) 0.82(-8.36) 0.87(-13.76) 0.97(-28.33) 0.95(-40.02) |
