# Supplementary material for: Validation of reference genes for use in untreated bovine fibroblasts
Source: Sci Rep. 2021 May 13;11:10253. doi: 10.1038/s41598-021-89657-8 (PMC8119449; doi:10.1038/s41598-021-89657-8)
Supplement: Supplementary file 3 — Supplementary Table S3. [file 41598_2021_89657_MOESM3_ESM.docx]

Validation of reference gene for use in untreated bovine fibroblasts.

Toorani T., Mackie P. M. & Mastromonaco G. F.

**Supplementary Table S3 - Candidate RG co-regulation assessment - ranked highest to lowest.** Candidate reference gene co-regulation evaluated using the Database of Gene Co-Regulation (dGCR; [www.dGCR.org](http://www.dGCR.org)), ranked in descending order.

| **Reference Gene** | **Co-regulated RG in top 150** | **dGCR score** |
| --- | --- | --- |
| HSP90AB1 | YWHAZ | -1625.23 |
| YWHAZ | HSP90AB1 | -1625.23 |
| RPL13A | RPS18 | -1255.01 |
| RPS18 | RPL13A | -1255.01 |
| ACTB | UBC | -750.24 |
| UBC | ACTB | -750.24 |
| ACTB | YWHAZ | -628.04 |
| YWHAZ | ACTB | -628.04 |
| GAPDH | HSP90AB1 | -603.08 |
| HSP90AB1 | GAPDH | -603.08 |
| GAPDH | RPL13A | -524.53 |
| RPL13A | GAPDH | -524.53 |
| RPS18 | B2M | -520.15 |
| HSP90AB1 | RPL13A | -501.57 |
| RPL13A | HSP90AB1 | -501.57 |
| GAPDH | PPIA | -494.52 |
| PPIA | GAPDH | -494.52 |
| GAPDH | UBC | -492.43 |
| UBC | GAPDH | -492.43 |
| ACTB | PPIA | -485.79 |
| PPIA | ACTB | -485.79 |
| PPIA | B2M | -460.51 |
| UBC | B2M | -448.84 |
| UBC | RPL13A | -433.13 |
| GAPDH | RPS18 | -416.5 |
| RPS18 | GAPDH | -416.5 |
| ACTB | B2M | -403.05 |
| PPIA | YWHAZ | -380.28 |
| PPIA | RPS18 | -376.91 |
| PPIA | UBC | -366.98 |
| UBC | PPIA | -366.98 |
| UBC | HSP90AB1 | -363.31 |
| PPIA | RPL13A | -358.36 |
| PPIA | HSP90AB1 | -333.81 |
| UBC | RPS18 | -328.04 |
| UBC | YWHAZ | -313.55 |
| HPRT1 | YWHAZ | -307.91 |
| ACTB | SDHA | -265.22 |
| SDHA | ACTB | -265.22 |
