# Supplementary material for: Validation of reference genes for use in untreated bovine fibroblasts
Source: Sci Rep. 2021 May 13;11:10253. doi: 10.1038/s41598-021-89657-8 (PMC8119449; doi:10.1038/s41598-021-89657-8)
Supplement: Supplementary file 4 — Supplementary Table S4. [file 41598_2021_89657_MOESM4_ESM.docx]

Validation of reference gene for use in untreated bovine fibroblasts.

Toorani T., Mackie P. M. & Mastromonaco G. F.

|  | **Excel plug-in** | | **R** |
| --- | --- | --- | --- |
| **Gene name** | **Stability value** | **Standard error** | **GroupSD** |
| HMBS | 0.0561 | 0.0228 | 0.08 |
| GUSB | 0.0587 | 0.0232 | 0.08 |
| TBP | 0.0615 | 0.0237 | 0.09 |
| ACTB | 0.0674 | 0.0249 | 0.1 |
| RPL13A | 0.0771 | 0.027 | 0.11 |
| PPIA | 0.0821 | 0.0281 | 0.12 |
| SDHA | 0.0825 | 0.0282 | 0.12 |
| GAPDH | 0.1098 | 0.0349 | 0.16 |
| YWHAZ | 0.1186 | 0.0372 | 0.17 |
| SF3A1 | 0.1226 | 0.0383 | 0.18 |
| UBC | 0.1439 | 0.0439 | 0.21 |
| RPS18 | 0.1494 | 0.0454 | 0.22 |
| HSP90AB1 | 0.1508 | 0.0458 | 0.22 |
| RAD50 | 0.1611 | 0.0486 | 0.23 |
| HPRT1 | 0.2365 | 0.0695 | 0.34 |
| B2M | 0.2593 | 0.076 | 0.37 |
|  |  |  |  |

**Supplementary Table S4 - NormFinder output.** NormFinder software results using both the Microsoft Excel plug-in and R versions.
