# Supplementary material for: Validation of reference genes for use in untreated bovine fibroblasts
Source: Sci Rep. 2021 May 13;11:10253. doi: 10.1038/s41598-021-89657-8 (PMC8119449; doi:10.1038/s41598-021-89657-8)
Supplement: Supplementary file 5 — Supplementary Table S5. [file 41598_2021_89657_MOESM5_ESM.docx]

Validation of reference gene for use in untreated bovine fibroblasts.

Toorani T., Mackie P. M. & Mastromonaco G. F.

**Supplementary Table S5 - RefFinder median vs. mean Ct results.** RefFinder software results using median and mean Ct values. As RefFinder does not specify input requirements, results derived from the use of median and mean Ct values were compared.

|  | **Median Ct** | | | | | **Mean Ct** | | | | |
| --- | --- | --- | --- | --- | --- | --- | --- | --- | --- | --- |
| Rank | Overall | geNorm (stability value) | NormFinder (stability value) | BestKeeper (standard deviation [± Ct]) | ΔCt (average of standard deviation) | Overall | geNorm (stability value) | NormFinder (stability value) | BestKeeper (standard deviation [± Ct]) | ΔCt (average of standard deviation) |
| 1 | GUSB (1.682) | GUSB (0.098) | HMBS (0.081) | TBP (0.184) | GUSB (0.2) | GUSB (1.414) | GUSB (0.097) | GUSB (0.079) | TBP (0.182) | GUSB (0.196) |
| 2 | HMBS (2.59) | RPL13A (0.098) | GUSB (0.085) | SDHA (0.192) | ACTB (0.205) | TBP (3.13) | RPL13A (0.097) | HMBS (0.080) | SDHA (0.192) | ACTB (0.199) |
| 3 | TBP (2.913) | HMBS (0.118) | TBP (0.089) | RPL13A (0.199) | HMBS (0.205) | RPL13A (3.224) | SDHA (0.115) | ACTB (0.088) | RPL13A (0.201) | HMBS (0.203) |
| 4 | RPL13A (2.943) | SDHA (0.133) | ACTB (0.097) | GUSB (0.205) | TBP (0.207) | HMBS (3.31) | HMBS (0.123) | TBP (0.090) | GUSB (0.206) | TBP (0.207) |
| 5 | ACTB (4.356) | ACTB (0.142) | RPL13A (0.111) | HMBS (0.219) | RPL13A (0.213) | SDHA (3.5) | ACTB (0.134) | SDHA (0.102) | HMBS (0.224) | SDHA (0.209) |
| 6 | SDHA (4.45) | TBP (0.147) | PPIA (0.119) | HSP90AB1 (0.231) | PPIA (0.215) | ACTB (4.054) | TBP (0.140) | RPL13A (0.112) | GAPDH (0.231) | RPL13A (0.212) |
| 7 | PPIA (7.565) | PPIA (0.155) | SDHA (0.119) | GAPDH (0.231) | SDHA (0.219) | GAPDH (8.132) | YWHAZ (0.153) | PPIA (0.139) | HSP90AB1 (0.236) | PPIA (0.222) |
| 8 | GAPDH (7.737) | GAPDH (0.164) | GAPDH (0.158) | RPS18 (0.241) | GAPDH (0.24) | PPIA (8.607) | PPIA (0.161) | YWHAZ (0.161) | RPS18 (0.242) | YWHAZ (0.236) |
| 9 | YWHAZ (10.051) | YWHAZ (0.171) | YWHAZ (0.171) | ACTB (0.247) | YWHAZ (0.244) | YWHAZ (8.736) | GAPDH (0.169) | GAPDH (0.166) | ACTB (0.247) | GAPDH (0.243) |
| 10 | HSP90AB1 (10.277) | SF3A1 (0.179) | SF3A1 (0.177) | B2M (0.252) | SF3A1 (0.248) | SF3A1 (10.466) | SF3A1 (0.177) | SF3A1 (0.180) | B2M (0.250) | SF3A1 (0.248) |
| 11 | SF3A1 (10.466) | HSP90AB1 (0.189) | UBC (0.208) | UBC (0.268) | UBC (0.272) | HSP90AB1 (10.681) | HSP90AB1 (0.188) | UBC (0.206) | UBC (0.271) | UBC (0.27) |
| 12 | RPS18 (10.843) | RPS18 (0.199) | RPS18 (0.216) | SF3A1 (0.277) | RPS18 (0.275) | RPS18 (11.062) | UBC (0.197) | RPS18 (0.214) | SF3A1 (0.272) | RPS18 (0.273) |
| 13 | UBC (11.469) | UBC (0.206) | HSP90AB1 (0.217) | PPIA (0.288) | HSP90AB1 (0.277) | UBC (11.242) | RPS18 (0.204) | HSP90AB1 (0.217) | YWHAZ (0.299) | HSP90AB1 (0.275) |
| 14 | B2M (14.226) | RAD50 (0.215) | RAD50 (0.232) | YWHAZ (0.31) | RAD50 (0.279) | B2M (14.226) | RAD50 (0.215) | RAD50 (0.244) | PPIA (0.301) | RAD50 (0.285) |
| 15 | RAD50 (14.244) | HPRT1 (0.233) | HPRT1 (0.341) | RAD50 (0.325) | HPRT1 (0.367) | RAD50 (14.244) | HPRT1 (0.230) | HPRT1 (0.321) | RAD50 (0.328) | HPRT1 (0.347) |
| 16 | HPRT1 (15.244) | B2M (0.254) | B2M (0.374) | HPRT1 (0.349) | B2M (0.403) | HPRT1 (15.244) | B2M (0.251) | B2M (0.368) | HPRT1 (0.341) | B2M (0.397) |
